# Clinical Lectures on Diseases of the Nervous System

**Published:** 1895-12

**Authors:** 


Clinical Lectures on Diseases of the Nervous System. Delivered at the National Hospital for the Paralysed and Epileptic, London. By W. R. Gowers, M. D., F. R. S., Physician to the Hospital; Consulting Physician to University College Hospital, etc. Octavo, pp. 279. Price, $2.00. Philadelphia: P. Blakiston, Son & Co., 1012 Walnut street. 1895.

The publication of clinical lectures in book form must always find justification either from the rarity of the case, or else from the charm of method of presenting old and familiar subjects.

It is the latter which makes this book delightful reading in leisure moments. One does not read it to learn new facts, but to study the method of presenting difficult subjects in what seems to us the most effective way. In every lecture may be found, scattered here and there, broad principles applicable to every physician's work, and useful in any lecture on any subject. As these add so much to the value of the book we are constrained to make a few isolated extracts:

It is well for a teacher, if he can, to resist the attraction of the new and it is always unwise for him to hesitate to inculcate that which is old merely because it is old. Truth and novelty are by no means necessarily associated.

The teacher should remember that to neglect to repeat is an unpardonable sin. The unpardonable sin of the student is to say yes when he ought to say no, to say that he saw a thing, that he heard a thing, that he felt a thing, that he understood a thing when he did not.

Never learn a diagnostic rule, indeed, never accept any general assertion without also endeavoring to ascertain on what the rule depends or the assertion rests. Unreasoned conclusions are the bane of students. Fix the assertions by their evidence, the rules by their reasons, and not only do they remain, but they take root and they become part of your real knowledge and a source of increasing power.

It is true of ourselves and it is true of our patients, that next to knowing what can be done and how to do it, the most important thing is to know what cannot be done. Sad is the waste of time and money caused by efforts to attain that which cannot be. Sadder still is the waste of hope—hope created by baseless expectation—and the destruction of it that we call disappointment; disappointment that would not be were it not for the anticipations that have no justification.

Let me at the outset warn you against the frequent error of thinking that a rare disease will only yield you knowledge which will be rarely needed. It is not so. Diseases which are uncommon cannot be studied without considering common facts and without giving increased ability to recognise diseases which are often met with.

In the postscript to the last lecture, the honesty of purpose of Professor Gowers is thus clearly shown:

The lecture is left as it was delivered. Although many statements have been contradicted by the later facts of the case, it is often useful thus to perceive such traversing of inference and fact, because it discloses the limits of our power of discerning that which is and of forecasting that which is to be.

The publishers are to be congratulated on their part of the work, for they have presented to the profession a library book in its truest sense. J. W. P.

